# Global comparative transcriptomes uncover novel and population-specific gene expression in esophageal squamous cell carcinoma

**DOI:** 10.1186/s13027-023-00525-8

**Published:** 2023-08-28

**Authors:** Amal Alotaibi, Veerendra P. Gadekar, Pranav Swaroop Gundla, Sumana Mandarthi, Nidhi Jayendra, Asna Tungekar, B. V. Lavanya, Ashok Kumar Bhagavath, Mary Anne Wong Cordero, Janne Pitkaniemi, Shaik Kalimulla Niazi, Raghavendra Upadhya, Asmatanzeem Bepari, Prashantha Hebbar

**Affiliations:** 1 Basic Science Department, College of Medicine, Princess Nourah bint Abdulrahman University, Riyadh, Saudi Arabia; 2 Mbiomics LLC, 16192 Coastal Highway, Lewes, DE 19958 USA; 3 Department of Cellular and Molecular Biology, University of Texas Health Science Center, Tyler, TX USA; 4 Finnish Cancer Registry, Unioninkatu 22, 00130 Helsinki, Finland; 5 Department of Public Health, Faculty of Medicine, University of Helsinki, Helsinki, Finland; 6 Department of Preparatory Health Sciences, Riyadh Elm University, Riyadh, Saudi Arabia; 7 Manipal Center for Biotherapeutics Research, Manipal Academy of Higher Education, Manipal, India; 8 Meta Biosciences Pvt Ltd, Manipal-GOK Bioincubator, Manipal, India

**Keywords:** Novel genes in ESCC, CHRM3, CREG2, H2AC6, ENDOU, FHL1, Esophagus cancer, ESCC, Toxicogenomics

## Abstract

**Background:**

Esophageal squamous cell carcinoma (ESCC) has a poor prognosis and is one of the deadliest gastrointestinal malignancies. Despite numerous transcriptomics studies to understand its molecular basis, the impact of population-specific differences on this disease remains unexplored.

**Aims:**

This study aimed to investigate the population-specific differences in gene expression patterns among ESCC samples obtained from six distinct global populations, identify differentially expressed genes (DEGs) and their associated pathways, and identify potential biomarkers for ESCC diagnosis and prognosis. In addition, this study deciphers population specific microbial and chemical risk factors in ESCC.

**Methods:**

We compared the gene expression patterns of ESCC samples from six different global populations by analyzing microarray datasets. To identify DEGs, we conducted stringent quality control and employed linear modeling. We cross-compared the resulting DEG lists of each populations along with ESCC ATLAS to identify known and novel DEGs. We performed a survival analysis using The Cancer Genome Atlas Program (TCGA) data to identify potential biomarkers for ESCC diagnosis and prognosis among the novel DEGs. Finally, we performed comparative functional enrichment and toxicogenomic analysis.

**Results:**

Here we report 19 genes with distinct expression patterns among populations, indicating population-specific variations in ESCC. Additionally, we discovered 166 novel DEGs, such as ENDOU, SLCO1B3, KCNS3, IFI35, among others. The survival analysis identified three novel genes (CHRM3, CREG2, H2AC6) critical for ESCC survival. Notably, our findings showed that ECM-related gene ontology terms and pathways were significantly enriched among the DEGs in ESCC. We also found population-specific variations in immune response and microbial infection-related pathways which included genes enriched for HPV, Ameobiosis, Leishmaniosis, and Human Cytomegaloviruses. Our toxicogenomic analysis identified tobacco smoking as the primary risk factor and cisplatin as the main drug chemical interacting with the maximum number of DEGs across populations.

**Conclusion:**

This study provides new insights into population-specific differences in gene expression patterns and their associated pathways in ESCC. Our findings suggest that changes in extracellular matrix (ECM) organization may be crucial to the development and progression of this cancer, and that environmental and genetic factors play important roles in the disease. The novel DEGs identified may serve as potential biomarkers for diagnosis, prognosis and treatment.

**Supplementary Information:**

The online version contains supplementary material available at 10.1186/s13027-023-00525-8.

## Introduction

Esophageal cancer is the eighth most commonly diagnosed cancer, accounting for 3% of all cancer cases and the sixth most common cause of death from cancer. The GLOBOCAN 2020 survey estimated a global increase in esophageal cancers with 604,000 new cases and 544,000 deaths in 2020. New estimation predicts that new cases will increase by about 1 million by 2040 [1]. Of the two histological types of esophageal cancers, 85% of the cases had esophageal squamous cell carcinoma and 14% had esophageal adenocarcinoma [1].

Striking differences were observed in the incidence, clinicopathological features, treatment efficacy, and overall prognosis of ESCC between geographical populations [2]. The highest incidence of ESCC is seen in Eastern Asia and Southern and Eastern Africa whereas the lowest is observed in Western Africa and Central America regions. Black patients had been diagnosed at the median age of 63 whereas Non-Hispanic Whites and Asian patients had 68 years as the median age at diagnosis. Similarly, survival differences were also observed between the ethnic groups. Black patients had the lowest while Non-Hispanic Whites had the better survival of ESCC [2].

It is important to note that specific risk factors for certain diseases can vary depending on the geographic region due to region-specific lifestyle, cultural, and environmental factors [3]. For example, salted meat consumption, alcohol intake, and smoking behavior are known risk factors for ESCC in Uganda [4]. In India, tobacco use is a common risk factor for ESCC, with approximately 34.6% of adults consuming tobacco through cigarettes or chewing [5]. Some malignancies are prone to the onset or progression because of trace element deficit or excess [6]. Trace element imbalance in the soils of South Africa and West Asia [3] showed high incidence and prevalence for ESCC. Dietary zinc deficiency is known to increase the risk of ESCC [7]. Zinc deficiency or excess has many carcinogenic impacts on cell growth, DNA repair, mutagenesis, apoptosis, DNA synthesis, and differentiation, and the overall balance of cellular antioxidants [6]. Similarly, selenium and zinc serum levels have been linked to the incidence of gastroesophageal cancers in West Asia [8]. In addition, frequent drinking of hot Arabic coffee in the Al-Qaseem region of Saudi Arabia [9], consumption of hot green tea in East Asia, and betel quid/gutka chewing in regions of South and Southeast Asia [10] are associated with the development of ESCC.

There is a growing understanding of the link between gut microbiota and human health [11]. Maintaining homeostasis and good health depends on the intestinal tract microbiota. Further, several types of cancers were initiated and progressed by the microbiome [12]. An imbalance of some species may contribute to the initiation and progression of tumors by harming DNA structure, generating metabolites that promote tumor growth, and inhibiting the immune response against tumors. About 50% of the ESCC tumors had human papillomavirus (HPV) infection in the southeastern region of Poland [13]. Studies have shown geographical region is an important factor for HPV prevalence in ESCC [14, 15].

Besides regional-specific risk factors, there exists a difference in genetic makeup among the global human populations by polymorphisms and their linkage disequilibrium [16]. Several Mendelian Randomization studies using genetic data have revealed causal associations between environmental factors and disease traits including cancers [17, 18]. Increasing evidence shows that environmental and lifestyle factors influence epigenetic changes. Changes in global epigenetic signatures, together with genetic alterations, are driving events in several types of cancer including ESCC [19, 20]. Hence, alteration in epigenetic signatures by region-specific risk factors and their interaction with population-specific genetic determinants may result in population-specific gene expression dysregulation and gene network perturbation in ESCC.

Although it is well-established that cancer causation involves multiple factors and gene-environment interactions [3, 21], to the best of our knowledge, no studies have attempted a comparative analysis between different populations to highlight the molecular connections with the risk factors. In this study, we conducted a comprehensive analysis of ESCC expression profiles from populations of various geographical regions, including Chinese, Japanese, Taiwanese, African American, Brazilian, and European populations. By examining the extent of shared gene perturbation across these populations, we aim to gain a better understanding of population-specific risk factors, diagnosis, prognosis, and treatment of ESCC. This systematic analysis will provide valuable insights into the molecular etiology of ESCC and help inform future research efforts in this field.

## Materials and methods

### Dataset

We collected protein-coding mRNA array expression datasets of human ESCC tissues and cell lines from—(1) Gene Expression Omnibus (NCBI-GEO) [22], (2) EBI ArrayExpress [23] and (3) All of the gene expression (AOE) [24]. We selected only those datasets where the studies compared the normal and tumor conditions. For the selected datasets, we inferred their origin population based on the description in the corresponding manuscript, which includes either the explicit mention of the study population or the location of the sample collection centers. If sample collection centers were spread across countries/continental hospitals, we labeled the population 'Unknown'.

### Data quality assessment

We used arrayQualityMetrics (v3.48.0) [25] in R statistical programming language (referred to as R in further instances) for the quality check of the datasets and to flag the potential outlier samples. Based on further manual inspection of the flagged outlier samples, we decided whether to include or exclude the samples from the downstream analysis. We disregarded the datasets that contained a potential sample mislabeling, class (control/tumor) imbalance issues or if retaining a dataset would decrease the overall common genes among the dataset in meta-analysis.

### Platform-specific background correction and normalization

Given that we selected the datasets generated from different platforms, we applied the platform-specific methods for background correction and normalization. We selected the appropriate normalization method that provided the normal data distribution of the data. For gene mapping of array probes, we used the array design files of platforms or Bioconductor annotation packages including AnnotationDBI (v1.58.0) [26] in R. Further multiple probes in each array were combined either by taking the mean or median values of probe intensities to represent the source gene.

### Differential gene expression of individual datasets

To discover differentially expressed genes in tumors compared to the normal samples in each dataset separately, we first generated the model matrix specifying the design (tumor ~ normal), followed by fitting the linear model to the design using lmFit() from the Limma Bioconductor package in R. Next, to perform empirical Bayesian moderation of the t-statistics we used the ebayes() from Limma that uses the distribution across genes to calculate a robust test statistic. We considered a gene as differentially expressed in the tumor compared to the normal samples if it showed a change in expression with absolute log 2 base fold change (log2FC) > 1.5 and adjusted p-value < 0.05.

### Meta-analysis for populations with multiple datasets

We performed the meta-analysis for the multiple datasets available for the same population using the MetaVolcanoR package (v1.10.0) available from the Bioconductor in R. The gene expression log2FC values, adjusted P-values, and 95% confidence intervals across multiple datasets were used as input data for the Random Effect Model (REM) approach implemented in the MetaVolcanoR. The random effect method takes into consideration the diversity present in multiple datasets by incorporating a statistical parameter that represents the inter-study variation, including clustering or dependence within a dataset, as well as varying relationships both within and between clusters [27]. REM summarizes the gene fold change taking into account the mean and variance that depends upon the study-specific estimates of the effect size. The genes consistently perturbed across all the studies were ranked based on the Topconfects approach [28] implemented within the package. Topconfects is a method for ranking by confidence bounds on the log fold change, based on the previously developed TREAT test by McCarthy and Smyth [29].

### Cross-comparative analysis of differentially expressed gene list

We compared the DEGs between populations using the ComplexHeatmap package for generating Upset plots in R. The common genes across at least two populations were compared against the ESCC ATLAS to identify if they were previously known to be associated with the ESCC etiology and if not, they were regarded as “novel DEGs” (in the rest of the manuscript). Similarly, we compared the DEGs unique among the populations against the ESCC ATLAS. The genes that did not map were discarded. For the downstream Functional Enrichment and Toxicogenomic analysis, we considered the common (including novel DEGs) and population-specific unique genes that mapped to ESCC ATLAS.

### Prioritization of novel genes

We used the Genotype-Tissue Expression portal (GTEx) portal bulk tissue-specific expression data or Human Protein Atlas (HPA) RNAseq normal tissue expression (tissue samples from 95 human individuals representing 27 different tissues) of Esophagus data to prioritize novel genes. The novel genes that were upregulated in our list but showed low or no expression in at least 3 different Esophagus tissues (Esophagus—Gastroesophageal Junction, Esophagus—Mucosa, and Esophagus—Muscular) in GTEx or HPA RNAseq, or vice versa were considered more important genes in the context to ESCC etiology.

### Survival analysis

To reveal the candidate gene contribution to the patient's survival, we performed overall survival prediction in an independent ESCC RNASeq dataset available at TCGA. We used TCGAbiolinks (v2.20.1) in R to access 90 ESCC tumors and 11 normal samples of the total 559 samples corresponding to Esophageal Cancer. Further, we used the survival package (v 0.4.9) and survminer package (v3.4.0) in R for survival analysis and for generating Kaplan–Meier survival plots respectively. For the genes that showed significant association with the survival of ESCC patients, we performed Cox proportional hazard analyses using the dysregulated and intact candidate genes and compared the distribution of log 2 base counts per million (log2CPM) values for these genes in the tumor and normal samples using t-test statistics.

### Functional and Toxicogenomic enrichment analysis

We performed functional enrichment for DEGs compared to all the expressed genes as background from each population separately using the ClusterProfiler (v4.4.4) [30] package in R. We examined the overrepresentation of GO terms and pathways using, gseGO(), gseKEGG(), gsePathway(), and gseWP() functions respectively. Where, the annotation data was used from the Gene Ontology [31], KEGG [32], Reactome [33], and WikiPathway [34] databases respectively. These functions compute the overrepresentation of terms based on the hypergeometric distribution test; the p-values were adjusted for the multiple testing in each case using the Benjamini-Hochberg (BH) procedure. The redundant enrichment terms were simplified using the simplify() function in the ClusterProfiler package. The GO terms and the Pathways were considered to be overrepresented among differentially expressed genes if the adjusted p-value for the enrichment analysis is < 0.05.

To infer the Toxicogenomic enrichment of our DEGs, we used the CTDquerier (v2.3.1) [35] package available from the Bioconductor in R. The CTDquerier retrieves the data from the Comparative Toxicogenomics Database (CTD), along with the evidence for direct gene-risk factor/drug associations. The CTD is a meticulously curated repository of interactions between chemicals, genes, and diseases extracted from scientific literature. In this study, we employed a network-based approach to calculate the inference score for genes and CTD terms relationships, specifically focusing on a chosen disease, such as ESCC. For this purpose, the background gene set comprised genes that were curated for ESCC in the CTD. A detailed information about inference score calculation is given in [36].

## Results

### Datasets selection

We used 14 published and 2 unpublished (namely, GSE23964 and GSE45168) datasets comprising clinical and expression information upon exhaustive search from publicly available data repositories. Detailed Information about the datasets with biological materials, sample size, microarray platforms, and sample collection centers used in each study is provided in Table 1. Based on our stringent quality check (refer to methods) we removed 4 data sets (GSE63941, GSE9982, GSE45168, GSE32424) (marked with the * symbol in Table 1). Based on the population inferences, the 12 datasets that we included in our study corresponded to either Chinese (5), Taiwanese (1), Japanese (1), African American (1), Brazilian (1), European (1), or unknown (2). An illustration of the workflow used in this study is provided in Additional file 1: Fig. S1.

**Table 1:** Datasets used in this study

Study	GSE ID	Biological Material (Sample Size)	Data generation platform	Sample collection center	Population
Hu et al. [92]	GSE20347	Tumor (17) and matched normal (17) esophageal tissue pairs	Affymetrix Human Genome U133A 2.0 Array	Shanxi Cancer Hospital in Taiyuan, Shanxi Province, PR China	Chinese^#^
Fujiwara et al. [Unpublished]	GSE23964	Normal (2) and tumor transfected in series of KYSE cell lines (14)	3D-Gene Human Oligo chip 25 k		Unknown
Chen et al. [93]	GSE70409	17 tumor and adjacent normal esophageal tissues	Phalanx Human OneArray	Kaohsiung Medical University Hospital, Taiwan	Taiwanese (inferred)
Aoyagi et al. [94]	GSE22954	Tumor (90) and normal (10) esophageal tissues	Affymetrix Human Genome U95A and U133 Arrays	Central Hospital or East Hospital at the National Cancer Center, Chiba, Japan	Japanese (inferred)
Yan et al. [95]	GSE33426	Tumor (59) and adjacent normal (12) esophageal tissues. GSE33426 had 18 common samples with GSE29001. They were removed	Affymetrix GeneChip human genome U133A 2.0 array	Shanxi Cancer Hospital and Institute, Taiyuan, Shanxi Province, China	Chinese (Taihang Mountain region)^#^
Wang et al. [96]	GSE26886	Tumor (19) and normal esophageal tissues (9)	Affymetrix Human Genome U133 Plus 2.0 Array	Charité Comprehensive Cancer Center, berlin, Germany	European (inferred)
Yan et al. [97]	GSE29001	Tumor (21), normal basal esophageal epithelium (12) and normal differentiated esophageal epithelium (12)	Affymetrix Genechip Human Genome U133A 2.0 Array	Shanxi Cancer Hospital and Institute, Taiyuan, Shanxi Province, China	Chinese^#^
Su et al. [37]	GSE23400	Tumor (53) and adjacent normal esophageal tissues (53)	Affymetrix GeneChip Human Genome U133A 2.0 Array	Shanxi Cancer Hospital in Taiyuan, Shanxi Province, People's Republic of China	Chinese (inferred)
Lee et al. [98]	GSE17351	Tumor (5) and adjacent normal esophageal tissues (5)	Affymetrix Genechip U133 plus v2.0 Array	Okayama University Hospital, Kitano Hospital, Japan and the Hospital of the University of Pennsylvania, USA	Unknown/Mixed
Nicolau-Neto et al. [99]	GSE75241	Tumor (15) and adjacent normal esophageal tissues (15)	Affymetrix Human Exon 1.0 ST array	Instituto Nacional de Câncer, Rio de Janeiro, Brazil	Brazilian (inferred)
Yang et al. [100]	GSE44021	Pairs of tumor (113) and normal (113) esophageal tissues	Affymetrix GeneChip Human Genome U133A 2.0 Arrays	Shanxi Cancer Hospital in Taiyuan, Shanxi Province, PR China	Chinese (inferred)
Erkizan et al. [101]	GSE77861	Tumor (7) and adjacent normal esophageal tissues (7)	Affymetrix Human Genome U133 Plus 2.0 Array		African American^#^
Tong et al. [102]	GSE32424 *	Tumor (7) and normal (5) esophageal tissues	Illumina Genome Analyzer IIx	Linzhou Cancer Hospital, Henan, China	Chinese (inferred)
Saito et al. [103]	GSE63941 *	22 ESCC cell lines and 4 isolated primary human esophageal fibroblasts	Affymetrix GeneChip Human Genome U133A array		Unknown
Shimokuni et al. [104]	GSE9982 *	20 KYSE human ESCC cell lines and 2 non-cancerous HEEC-1 esophageal epithelial cell lines	Codelink Uniset Human 20 K I Bioarray		Unknown
Zhu et al. [Unpublished]	GSE45168 *	Tumor (5) and adjacent normal (5) esophageal tissues	Agilent-026652 Whole Human Genome Microarray 4 × 44 K v2		Unknown

‘#’ population information explicitly mentioned in the study, ‘$’ population information inferred from the sample collection center described in the study, ‘*’ indicates these datasets have been removed based on QC

### Distribution of DEGs in ESCC among different populations

**Fig. 1:**
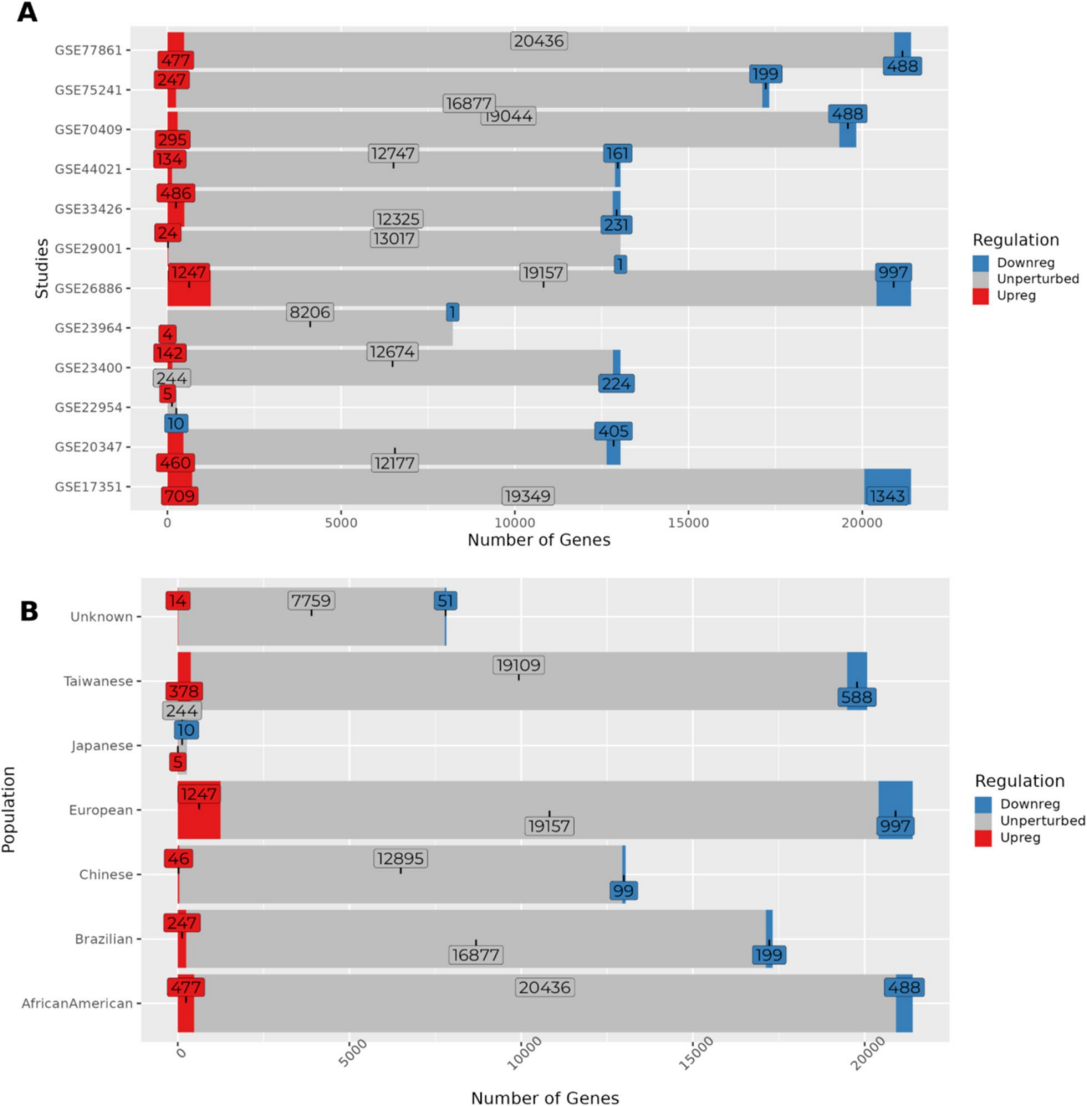
illustrates **A** Total Up-, Down-regulated and unperturbed genes in the 12 datasets used in this analysis. **B** Population-wise total gene counts (including meta-analysis of 5 Chinese datasets and 2 unknown population datasets). Note: log2FC > 1.5 | < -1.5 and adjusted p-value < 0.05 was used as a threshold for filtering the genes

In order to identify the DEGs in tumors compared to the normal samples among all the populations, we performed the differential gene expression analysis as discussed in the methods section. The observed and expected quantiles of the 12 datasets are shown in the QQ plot (Additional file 1: Fig. S2), indicates no bias or confounding factors in the results of analyzed datasets. The total number of DEGs in each dataset is shown in Fig. 1A.

We found on average 1155 ± 766 (Mean ± SD) DEGs in the European, African American, and Taiwanese populations, whereas 75 ± 65 (Mean ± SD) DEGs in the Chinese, Japanese, and Unknown populations. This was expected given in the latter populations, the data set included a lower number of genes. For the Chinese and unknown populations, we had multiple datasets. The total up- and down-regulated DEGs are shown in Fig. 1B.

### Comparative analysis of DEGs across populations

**Fig. 2:**
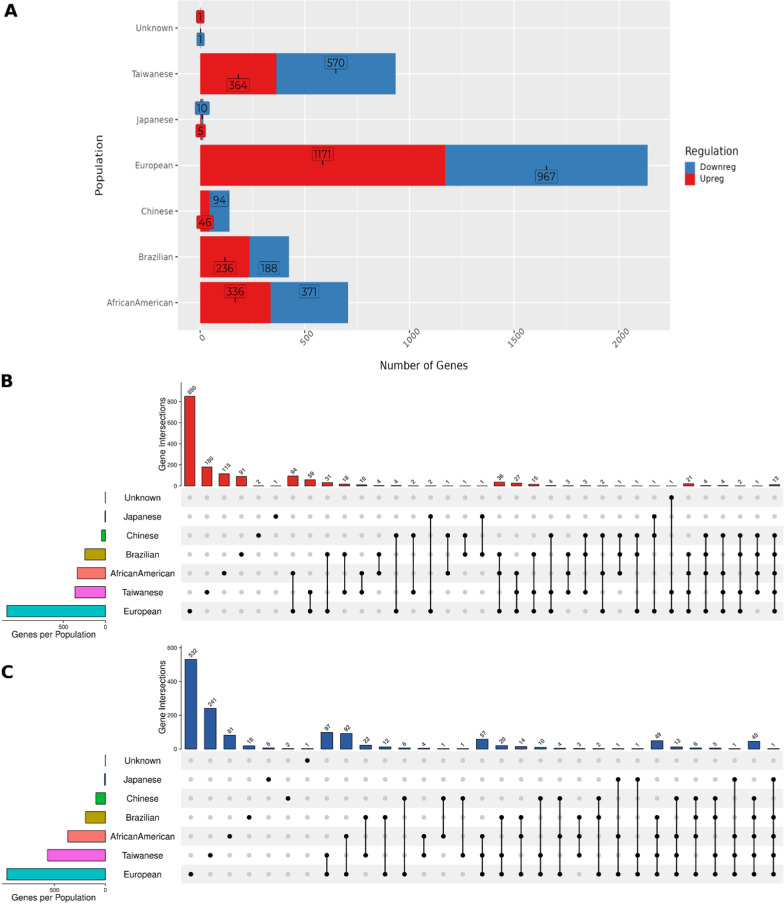
**A** Total up- and down-regulated genes in each population intersecting with ESCC ATLAS. Cross-comparison of up (**B**) and down-regulated (**C**) genes across populations

We identified 1442 DEGs of which 1423 (Additional file 2: Table S1) were concordant DEGs (expressed commonly up or down-regulated between the population), and the remaining 19 genes were discordant DEGs where the direction of gene expression (up/down) in at least one population is different compared to the rest. Among the 1423 concordant DEGs, we identified 1257 mapped to ESCC ATLAS. We have summarized the total DEGs across populations upon filtering against ESCC ATLAS in Fig. 2A. The common and unique Up- and Down-regulated genes across populations are shown in Fig. 2B and C respectively. Some of the interesting genes among these include—SASH1 (SAM and SH3 domain containing 1), a potential tumor suppressor with negative regulation of proliferation, and invasion of cancer cells. We identified it is significantly downregulated in African-American, Brazilian, European, Japanese, and Taiwanese populations in our analysis. Additionally, ESCC ATLAS catalogs a study that suggests SASH1 is downregulated in the Chinese population as well in the onset of ESCC [37],—BLNK (B Cell Linker is a leukocyte protein that contains SH2 domain) that is known to produce B-cell linker protein crucial for B-cell development [38]. We find this gene is downregulated in 5 populations except in Japanese.

**Fig. 3:**
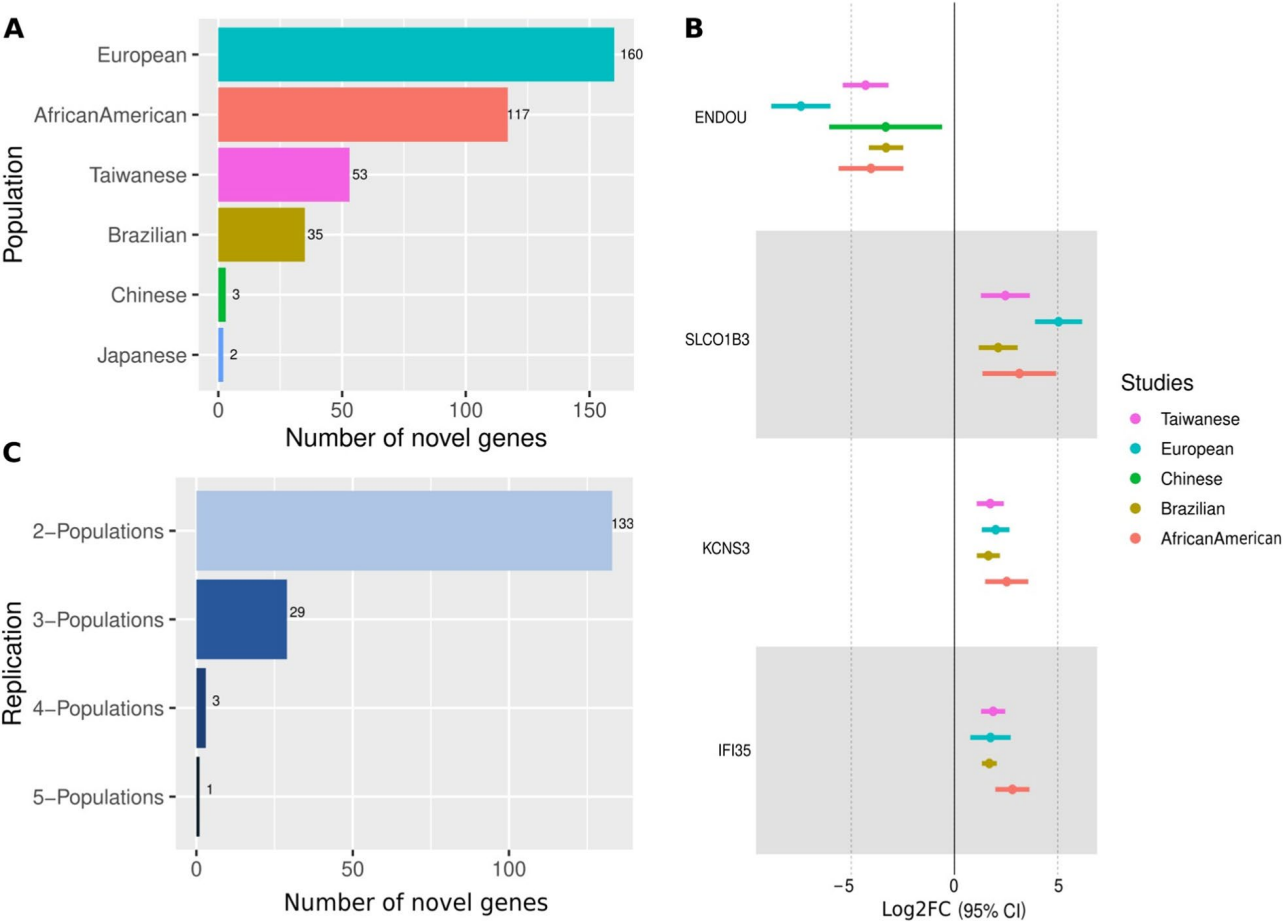
**A** Total Novel DEGs across populations, **B** Total Novel DEGs common between populations, **C** Expression distribution of 4 Novel DEGs across populations

We considered the remaining 166 DEGs out of 1423 concordant DEGs in our analysis as novel genes in the context of ESCC etiology because we did not find any prior evidence for their involvement in ESCC. To investigate the expression of these DEGs in healthy conditions, we compared our list of DEGs with the expression profile of genes in healthy Esophageal-Mucosa using GTEx or HPA RNAseq data. We discovered that 18 DEGs (ENDOU, ANKRD20A5P, CYP4F35P, CYSRT1, EPIST (also known as C5orf66-AS1), LCAL1, LINC02487, LOC100507221, LOC105376081, MUCL3 (also known as DPCR1), SELENOM, TENT5B (also known as FAM46B), TOX2, DSG1 (based on DSG1-AS1 expression), DEGS2, DOUXA2, KLK7, RNF39) exhibited an opposite expression trend in normal Esophagus tissues. This finding provides additional evidence for these genes to play a crucial role in the context of ESCC (Fig. 3).

To highlight a few, here are the novel DEGs that are concordant in at least 4 populations—(1) ENDOU (endonuclease, poly(U) specific), is significantly (fold change and p-value) downregulated in ESCC samples across 5 populations except in Japanese (where it was not included in the array), whereas, it is seen to be overexpressed in healthy Esophageal-Mucosa tissues in GTEx. ENDOU is known to encode for a protein with endoribonuclease activity that binds to the polyuridine-enriched single-stranded RNA. Interestingly, ENDOU in mice is identified as a regulator of activation-induced cell death. (2) KCNS3 (potassium voltage-gated channel modifier subfamily S member 3), is upregulated in at least four populations (see Table 2), but in healthy Esophageal-Mucosa listed in GTEx, it is lowly expressed. Interestingly, the knockdown of KCNS3 inhibits tumor cell proliferation in colon carcinoma and lung adenocarcinoma cell lines [39]. (3) SLCO1B3 (solute carrier organic anion transporter family member 1B3), is found to be upregulated in our analysis (see Table 2), however, it is not expressed in healthy Esophageal-Mucosa in GTEx data. Interestingly, the overexpression of SLCO1B3 in non‑small cell lung cancer cells is known to regulate the epithelial‑mesenchymal transition (EMT) related genes [40]. (4) IFI35 (interferon-induced protein 35), is upregulated in four different populations in our analysis (see Table 2), however, it is found to be lowly expressed in healthy Esophageal-Mucosa reported in GTEx. IFI35 is known to be involved in processes such as macrophage activation in immune response and positive regulation of defense response. The list of novel DEGs common in at least two populations is listed in Additional file 2: Table S2.

**Table 2:** List of Novel DEGs that are concordant in at least 4 populations

Gene	Log2FC	Adj. P-value	Population
ENDOU	− 4.04	4.60E-03	African American
ENDOU	− 3.32	3.39E-07	Brazilian
ENDOU	− 3.33	2.26E-03	Chinese
ENDOU	− 7.44	6.73E-09	European
ENDOU	− 4.31	3.17E-07	Taiwanese
IFI35	2.8	1.15E-03	African American
IFI35	1.68	3.64E-08	Brazilian
IFI35	1.74	6.53E-03	European
IFI35	1.87	7.53E-06	Taiwanese
KCNS3	2.52	6.41E-03	African American
KCNS3	1.63	2.37E-05	Brazilian
KCNS3	1.99	3.46E-05	European
KCNS3	1.73	1.23E-04	Taiwanese
SLCO1B3	3.14	3.01E-02	African American
SLCO1B3	2.11	5.75E-04	Brazilian
SLCO1B3	5.04	9.99E-08	European
SLCO1B3	2.46	1.94E-03	Taiwanese

We observed consistent downregulation of ENDOU in 5 population and upregulation of IFI35, KCNS3, and SLCO1B3 in 4 population

Discordant genes are particularly interesting because they point towards potential population-specific expression profiles of important genes in the ESCC etiology. Where up or down-regulation of specific genes could be either deleterious or advantageous for only a subset of the populations. In total, we identified 19 discordant DEGs (see Table 3 and Additional file 2: Table S3) across populations. Some interesting genes include—(1) FHL1 (Four and a half LIM domains protein 1) is upregulated in African American, Brazilian, and European whereas downregulated in Taiwanese. Overexpression of FHL1 is reported to inhibit the cell proliferation, colony formation potential, and expression of CDK4 and Cyclin D1 thereby negatively regulating the Wnt/β-catenin signaling pathway [41], (2) MYL9 (Myosin light chain 9) is upregulated in European but downregulated in the Taiwanese population. The overexpression of MYL9 is known to promote cell proliferation, invasion, migration, and angiogenesis [42]. Of these 19 genes, we did not find prior evidence for 6 genes (TPPP3, IGSF22, SMPX, TPTE2P1, RBPMS2, and TCEAL2) for their involvement in ESCC (Additional file 2: Table S4).

**Table 3:** List of 19 DEGs with discordant expression between populations

Gene	Population (Evidence from ESCC ATLAS)	Log2FC	Adj. P-value	Gene	Population (Evidence from ESCC ATLAS)	Log2FC	Adj. P-value
FHL1	African American	1.68	1.86E-02	TCEAL2	European	1.7	4.91E-02
FHL1	Brazilian	1.55	1.23E-04	TCEAL2	Taiwanese	− 2.11	1.04E-02
FHL1	European	1.51	3.16E-02	CNN1	European	2.01	1.20E-02
FHL1	Taiwanese (South Indian, Chinese)	− 1.69	1.10E-02	CNN1	Taiwanese (South Indian, Chinese)	− 2.95	2.25E-05
TPPP3	African American	3.32	4.64E-02	SYNM	European	1.56	1.83E-03
TPPP3	Chinese	− 1.5	5.92E-13	SYNM	Taiwanese (South Indian)	− 2.41	5.21E-04
IGSF22	African American	1.81	1.02E-03	PLN	European	2.31	1.10E-02
IGSF22	European	− 1.88	3.55E-03	PLN	Taiwanese (South Indian)	− 1.91	2.22E-02
KCND1	African American	− 1.78	3.12E-02	LYZ	Brazilian (Chinese)	1.68	5.76E-05
KCND1	European	2.01	5.35E-03	LYZ	European	− 2.09	2.57E-02
SMPX	African American	− 1.84	2.82E-02	ACTG2	Brazilian	1.51	1.90E-02
SMPX	European	1.61	1.78E-02	ACTG2	Taiwanese (South Indian)	− 2.5	1.01E-05
TPTE2P1	African American	− 1.6	1.69E-02	RBPMS2	European	1.82	4.69E-02
TPTE2P1	European	1.96	1.72E-03	RBPMS2	Taiwanese	− 1.77	1.55E-02
IL36G	African American (South Indian)	2.6	4.20E-02	FGF3	European	2.03	1.50E-02
IL36G	European	− 2.77	1.44E-02	FGF3	Taiwanese	− 1.7	4.32E-03
PCP4	European	2.16	4.35E-02	KIF26B	European (South Indian)	2.1	3.82E-05
PCP4	Taiwanese	− 2.52	1.36E-04	KIF26B	Taiwanese	− 1.86	2.35E-05
MYL9	European	1.61	2.10E-02	MYH11	European	1.93	7.33E-03
MYL9	Taiwanese (South Indian)	− 1.75	9.96E-03	MYH11	Taiwanese (Chinese)	− 2.91	7.20E-05

This table presents a list of 19 differentially expressed genes (DEGs) that exhibit discordant expression patterns between populations. It also includes evidence for the observed gene expression direction from the ESCC ATLAS

### Candidate genes associated with ESCC prognosis

**Fig. 4:**
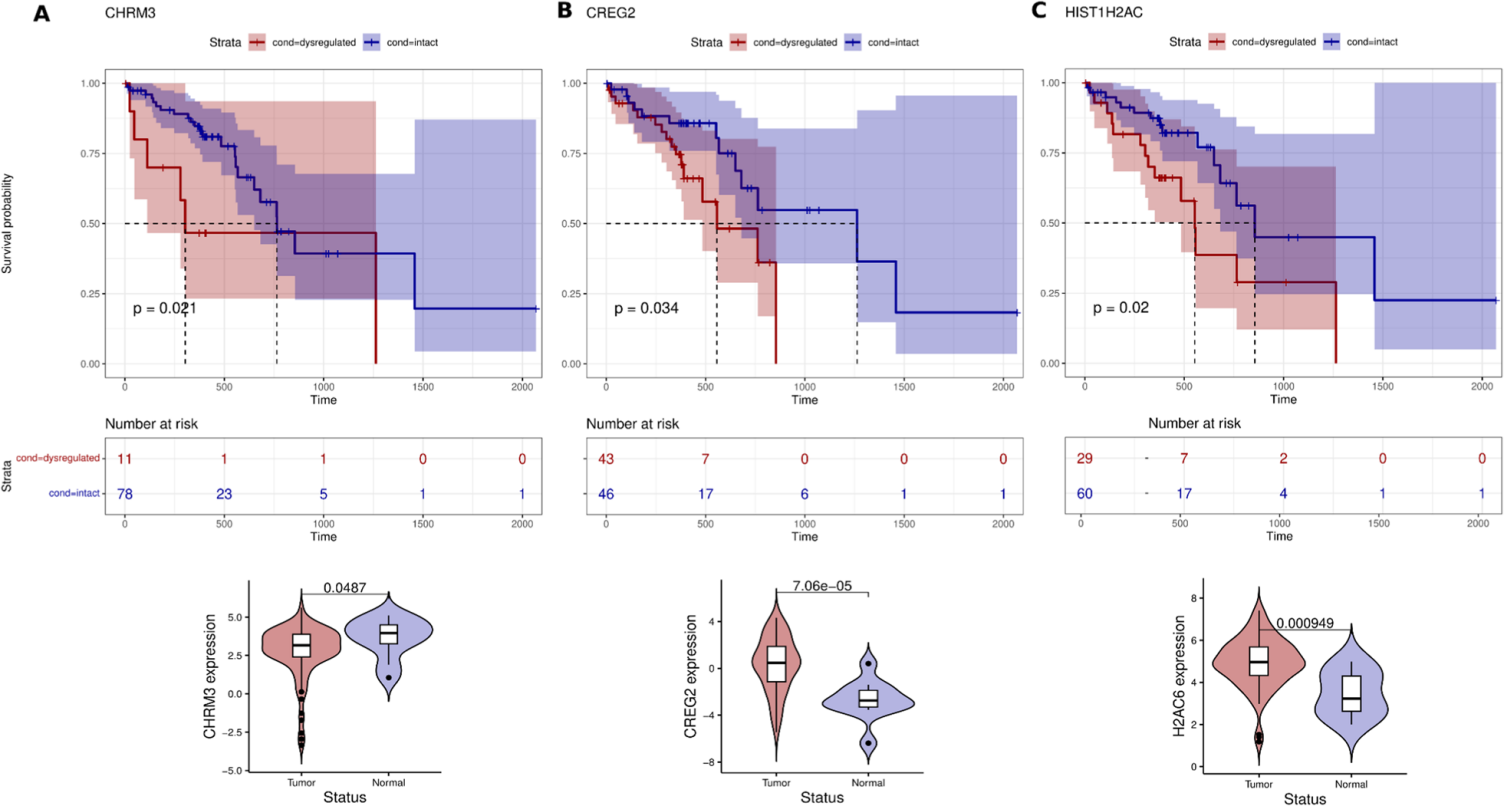
illustrates DEGs with significant effect on Survival and observed mean differences between normal-tumor samples for the genes **A** CHRM3, **B** CREG2 and **C** HIST1H2AC (alias H2AC6) in ESCC data from TCGA

In order to identify the candidate genes from our list of 166 novel DEGs in ESCC that could be potential prognostic markers in ESCC, we performed survival analysis (See “Methods”). Interestingly, we identified 3 genes—CHRM3, CREG2, and H2AC6 (alias HIST1H2AC) that were significantly correlated with overall survival in ESCC. The increased expression levels of CHRM3 and CREG2 were significantly associated with lower survival time; this was supported by the Hazard Ratio (HR) of 2.84 (p-value = 0.026) and 2.29 (p-value = 0.039) respectively. In the case of H2AC6 gene, its downregulation is significantly associated with lower survival time (HR = 2.35, p-value = 0.023) (Fig. 4). In our analysis we identified CHRM3, CREG2 to be upregulated in African American and Europeans, whereas, H2AC6 was downregulated in these populations. The expression profiles (up/down-regulation) of these three genes in TCGA RNA-seq samples were well correlated with the profiles observed in our datasets. To the best of our knowledge, these genes have not been discussed in the context of ESCC thus far. Given that they are differentially expressed in ESCC and show a significant impact on survival time, we suggest these three genes as candidate genes for ESCC prognosis.

### Gene ontology and pathway enrichment of DEGs

**Fig. 5:**
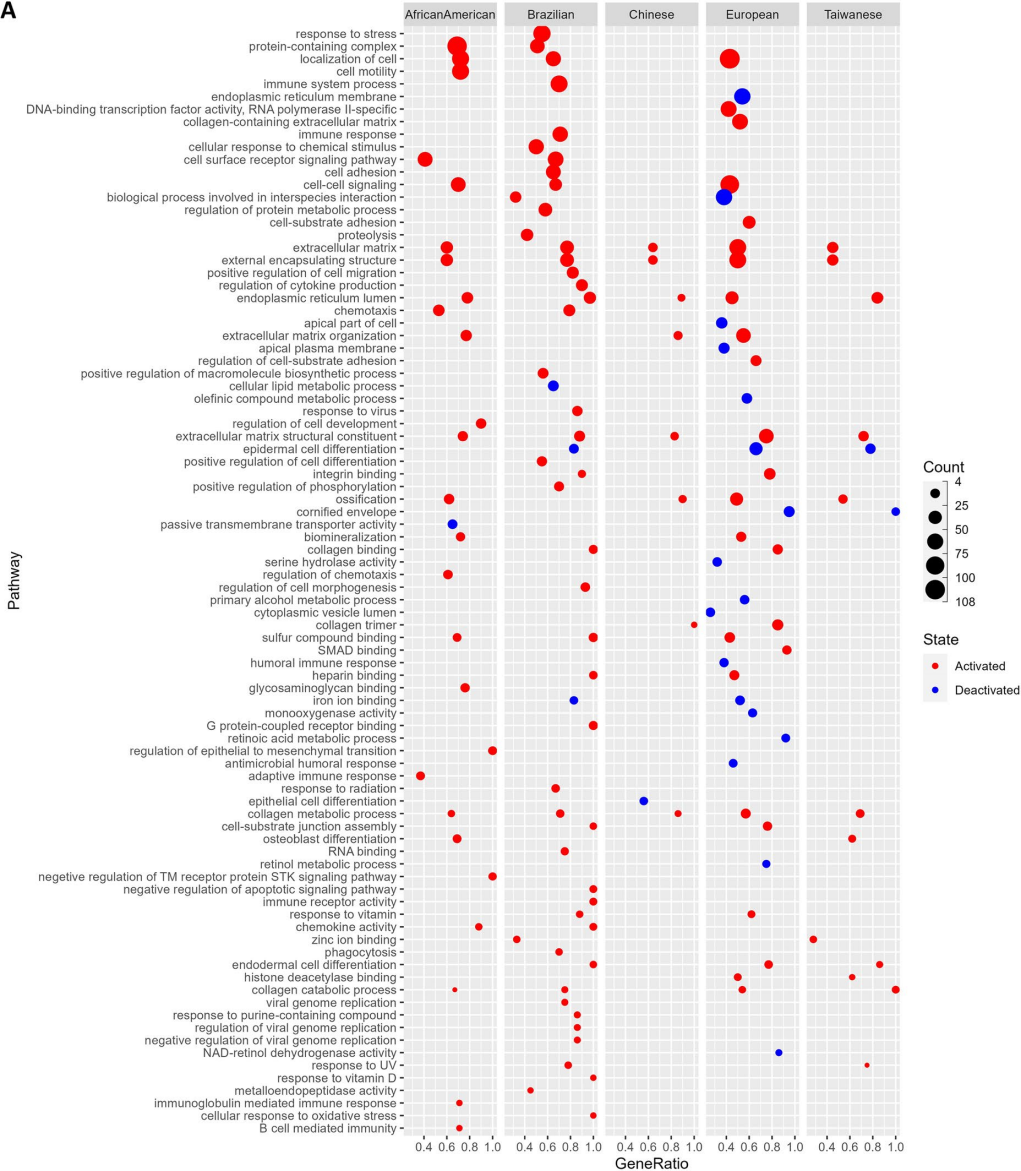

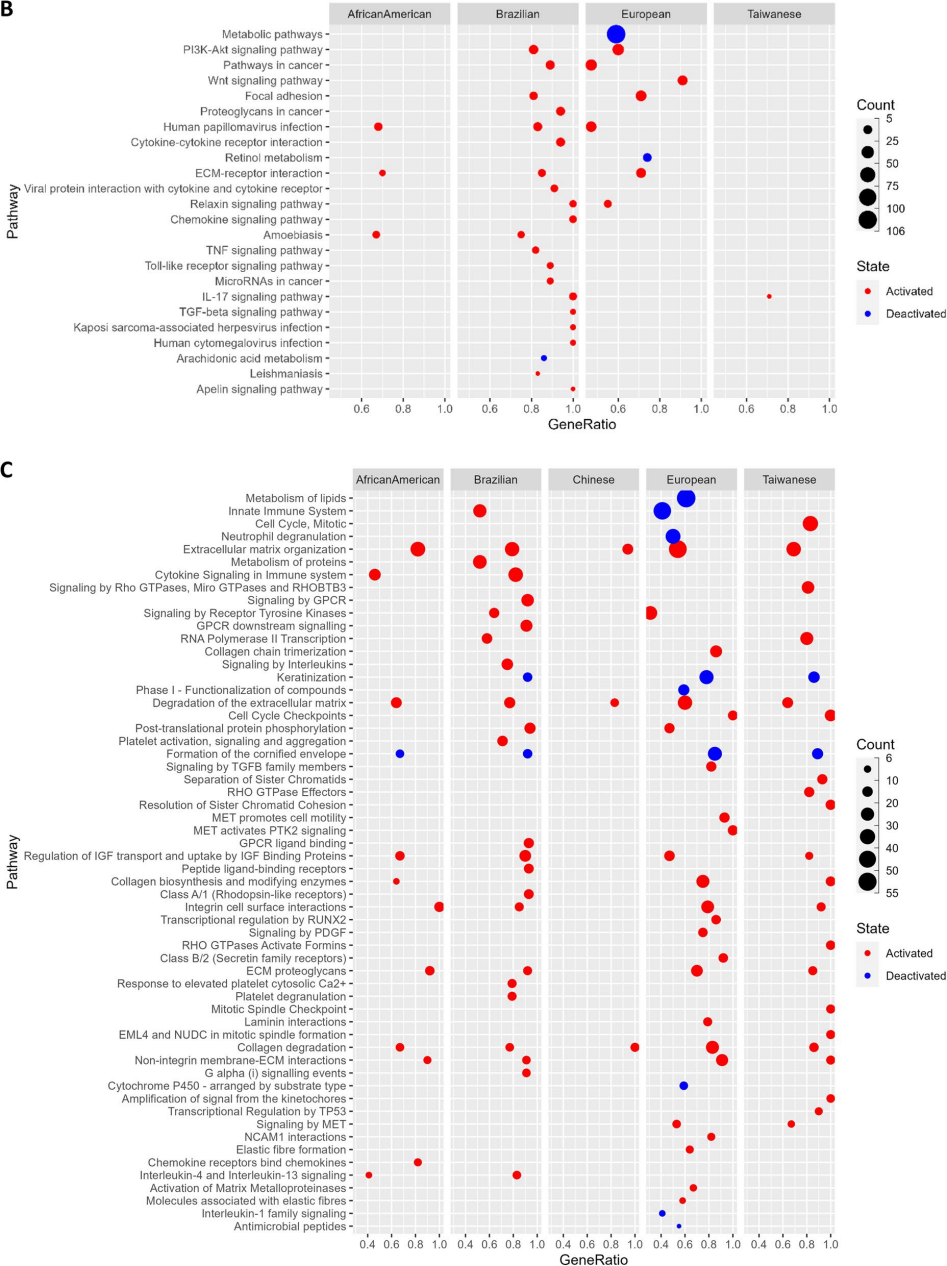

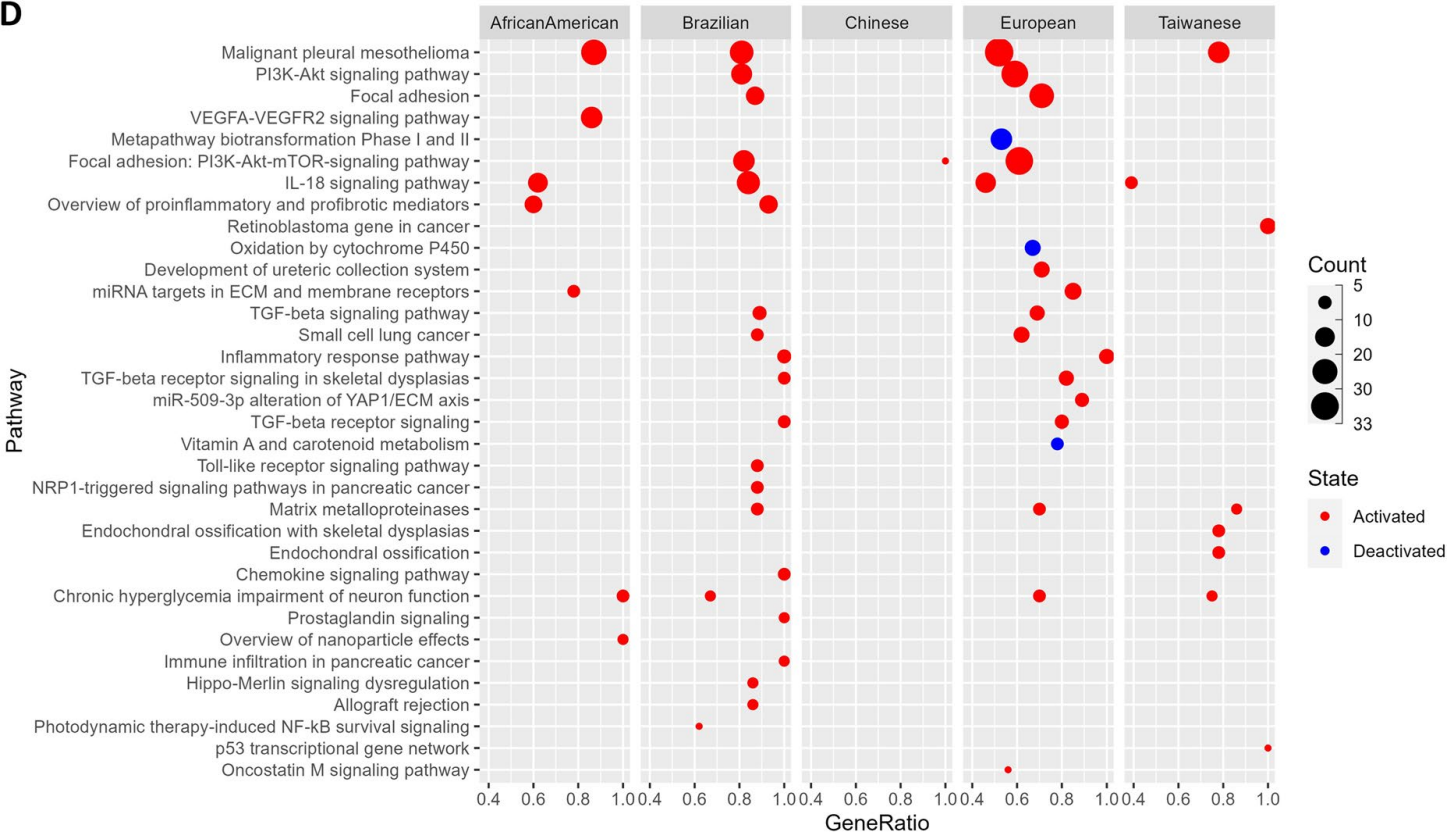
Overrepresented **A** GO terms—Biological Process, **B** Pathway terms from KEGG, **C** REACTOME, **D** Wiki pathways, among DEGs

In order to identify the potential functional role of the DEGs in our analysis, we performed functional enrichment analysis using the publicly available GO and pathway annotation data (See “Methods”). Interestingly, we found multiple GO terms and pathways (Additional file 2: Tables S5–S8) related to the Extra Cellular Matrix (ECM) organization, and its intricate molecular cascades were significantly overrepresented in our list of DEGs (Fig. 5). These included—Degradation of the extracellular matrix, collagen metabolic process (activated), cell-substrate junction assembly, epidermal and endodermal cell differentiations, cornified envelope (deactivation), Keratinization (deactivation), Focal adhesion: PI3K-Akt-mTOR signaling pathway (Activated), Integrin binding (Activated), Cell motility, Cell migration, and Cells localization, etc. This is in agreement with previous reports, where the ECM-based communication between cells and their surrounding microenvironment is discussed to affect the genesis and/or development of esophageal tumors [43, 44].

Another important enrichment term discovered was Ossification (Fig. 5A), a condition that involves the muscles becoming bruised and forming bone-like structures. Several ossification-related cases were reported in ESCC [45]. Ossifying tumors may arise in the skeleton, the viscera, or the soft tissues and are classified according to the tissue of origin and histological characteristics. We also identified several inflammatory response-related interleukin activations pathways to be overrepresented in our DEGs; these included—Interleukin (IL) -4 and IL-13 signaling, IL-17 signaling pathway, and IL-18 signaling pathway (Fig. 5B–D). The ESCC patients indeed were reported to show high IL-7R expression that potentially contributes to the ESCC progression by promoting the development of various malignant phenotypes [46]. We also identified the signaling pathways such as VEGFA-VEGFR2 (activated) signaling that regulates the cell migration, proliferation, and survival in the formation of new blood vessels to be overrepresented in our DEGs list in context to ESCC. In fact, blocking the VEGFA1-VEGFR2 signaling is a suggested therapy with potential benefits for patients with aggressive EC [47]. Similarly, the transforming growth factor beta (TGF-Beta) signaling pathway (activated) is overrepresented by our DEGs which involves many cellular processes, including cell growth, cell differentiation, cell migration, apoptosis, cellular homeostasis, and other cellular functions. Interestingly, one of the previous reports suggested that the inhibition of this pathway prevents ESCC-induced neoangiogenesis [48, 49].

We also identified the overrepresentation of Toll-like receptor signaling pathways that play crucial roles in the innate immune system by recognizing pathogen-associated molecular patterns derived from various microbes [50], which potentially involves the genes linked to Human Papilloma Virus which is suggested to contribute to ESCC in a high-risk population [51], such as African American based on our analysis. Other microbial infections include Amoebiasis in African American and Brazilian populations, Leishmaniasis, and Human Cytomegalovirus infection in Brazilian populations (Fig. 5B). Literature evidences support these plausible microbial infections in ESCC. For example (i) Infection of several species of Leishmania was observed with Squamous Cell Carcinoma in Brazil and Iran [52–54], and a long term infection of it suspected to induce DNA methylation alterations triggering tumorigenesis [52], (ii) CMV infection associated gastrointestinal tract lesion- esophagitis in a Japanese patient observed with a moderately differentiated squamous cell carcinoma [55] and reactivation of CMV infection among esophageal cancer patients undergoing chemotherapy in Japan [56], (iii) Cervical cancer patients in India [57] and colon cancer patients in India and Japan [58, 59] found with Amoebiasis. Further, *Entamoeba histolytica* infects epithelial cells expressing EhADH adhesin together with the EhCP112 cysteine protease and damage epithelium [60]. The genes overrepresented for microbial infections among respective populations is listed in Table 4.

**Table 4:** List of microbial infection terms seen activated across populations

Microbial infection terms	Normalized Enrichment score	Adjusted P-value	Genes	Population
Human papillomavirus infection	0.49	7.1E-04	COL1A2, COL4A1, COL4A2, COL6A3, CREB5, FN1, FZD2, HEY1, ISG15, LAMC2, PTGS2, SPP1, TNC	African American
Human papillomavirus infection	0.56	5.1E-05	AKT3, CCNA1, COL1A1, COL1A2, FN1, HEY1, ITGA5, ITGA6, LAMB3, LAMC1, LAMC2, PTGS2, SPP1, THBS1, TNC	Brazilian
Human papillomavirus infection	0.40	1.28E-05	CCNA1, COL1A2, COL4A1, COL4A2, COL4A6, COL6A3, COL9A3, FN1, FZD2, HES4, HEY1, ISG15, ITGA3, ITGA5, LAMB3, LAMC1, LAMC2, PTGS2, RELN, SPP1, THBS1, THBS2, THBS4, TNC	European
Amoebiasis	0.56	4.7E-04	COL1A2, COL3A1, COL4A1, COL4A2, CXCL1, CXCL8, FN1, IL6, LAMC2, PLCB4	African American
Amoebiasis	0.49	0.047	COL1A1, COL1A2, COL3A1, CXCL1, FN1, IL1B, LAMB3, LAMC1, LAMC2	Brazilian
Human cytomegalovirus infection	0.68	0.030	AKT3, CCL2, CCR1, GNA12, IL1B, PTGS2	Brazilian
Leishmaniasis	0.79	5.2E-03	C3, FCGR2A, FCGR3A, IL1B, PTGS2	Brazilian

### Toxicogenomic risk factors, and drug chemicals enrichment of DEGs

In order to investigate if our list of DEGs in ESCC was linked to any potential adverse effects that could result from factors such as exposure to environmental or chemical toxins we inferred their toxicogenomic enrichment from the Comparative Toxicogenomics Database (See Methods). We identified 11 genes such as PTGS2, SLC39A6, TAGLN, HMGN5, KRT17, FAT1, SERPINB3, ALDH2, ANXA1, SALL4, and TPM1 with evidence of direct association with the risk factors such as 4-Nitroquinoline-1-oxide, nitroso benzylmethylamine, Tobacco Smoke Pollution, and Zinc, and drug chemicals such as Cisplatin, diallyl trisulfide, Docetaxel, Fluorouracil, Mitomycin and Vinorelbine (Table 5). Of these interesting genes, SLC39A6 is identified as a therapeutic target in previous reports [61] for Ladiratuzumab Vedotin (a Zinc transporter ZIP6 binding agent) drug to treat triple-negative breast cancer and angiosarcoma. Polymorphisms in ALDH2 increased the risk of esophageal cancer with exposure to ethanol and cigarette smoking [62]. Sall4 is an essential regulator in cisplatin-induced apoptosis, and knockdown of Sall4 may restore cisplatin sensitivity in acquired resistant cells [63]. Similarly, FAT1 downregulation enhanced cisplatin resistance and stemness in ESCC [64]. Dietary Zinc known to modulate PTGS2 expression [65]. TPM1 is known to be downregulated by overexpression of miR-21 in inflammatory esophagus and tongue of Zinc deficient rat [66].

**Table 5:** List of DEGs directly interacting with risk factors or drug chemicals inferred from the comparative toxicogenomic database

Gene	Evidence type	Inference network	Inference score	Reference count	Population
PTGS2	Marker/mechanism	4-Nitroquinoline-1-oxide | Cisplatin | diallyl trisulfide | Docetaxel | Fluorouracil | Mitomycin | nitrosobenzylmethylamine | Tobacco Smoke Pollution | Vinorelbine | Zinc	9.41	14	African American, Brazilian, European
SLC39A6	Therapeutic (LADIRATUZUMAB VEDOTIN-Zinc transporter ZIP6 binding agent) for tripple -ve breast cancer and angiosarcoma	Tobacco Smoke Pollution | Zinc	4.9	7	African American
TAGLN	Marker/mechanism	Cisplatin | Fluorouracil | Tobacco Smoke Pollution	5.5	5	African American
HMGN5	Marker/mechanism	Cisplatin | Fluorouracil | Tobacco Smoke Pollution	8.74	5	African American, European
KRT17	Marker/mechanism	Cisplatin | Fluorouracil | nitrosobenzylmethylamine | Tobacco Smoke Pollution	10.22	6	African American, Brazilian, European, Chinese
FAT1	Marker/mechanism	Mitomycin | nitrosobenzylmethylamine | Tobacco Smoke Pollution	10.22	6	Brazilian, European
SERPINB3	Marker/mechanism	nitrosobenzylmethylamine | Tobacco Smoke Pollution | Zinc	9.69	8	Chinese, European, Taiwanese
ALDH2	Marker/mechanism	Cisplatin | nitrosobenzylmethylamine | Tobacco Smoke Pollution | Zinc	7.51	11	European
ANXA1	Marker/mechanism	Cisplatin | diallyl trisulfide | Fluorouracil | nitrosobenzylmethylamine | Tobacco Smoke Pollution | Zinc	13.15	11	European
SALL4	Marker/mechanism	Cisplatin | Tobacco Smoke Pollution	4.95	5	European
TPM1	Marker/mechanism	Cisplatin | Fluorouracil | Tobacco Smoke Pollution | Zinc	8.18	9	European

**Fig. 6:**
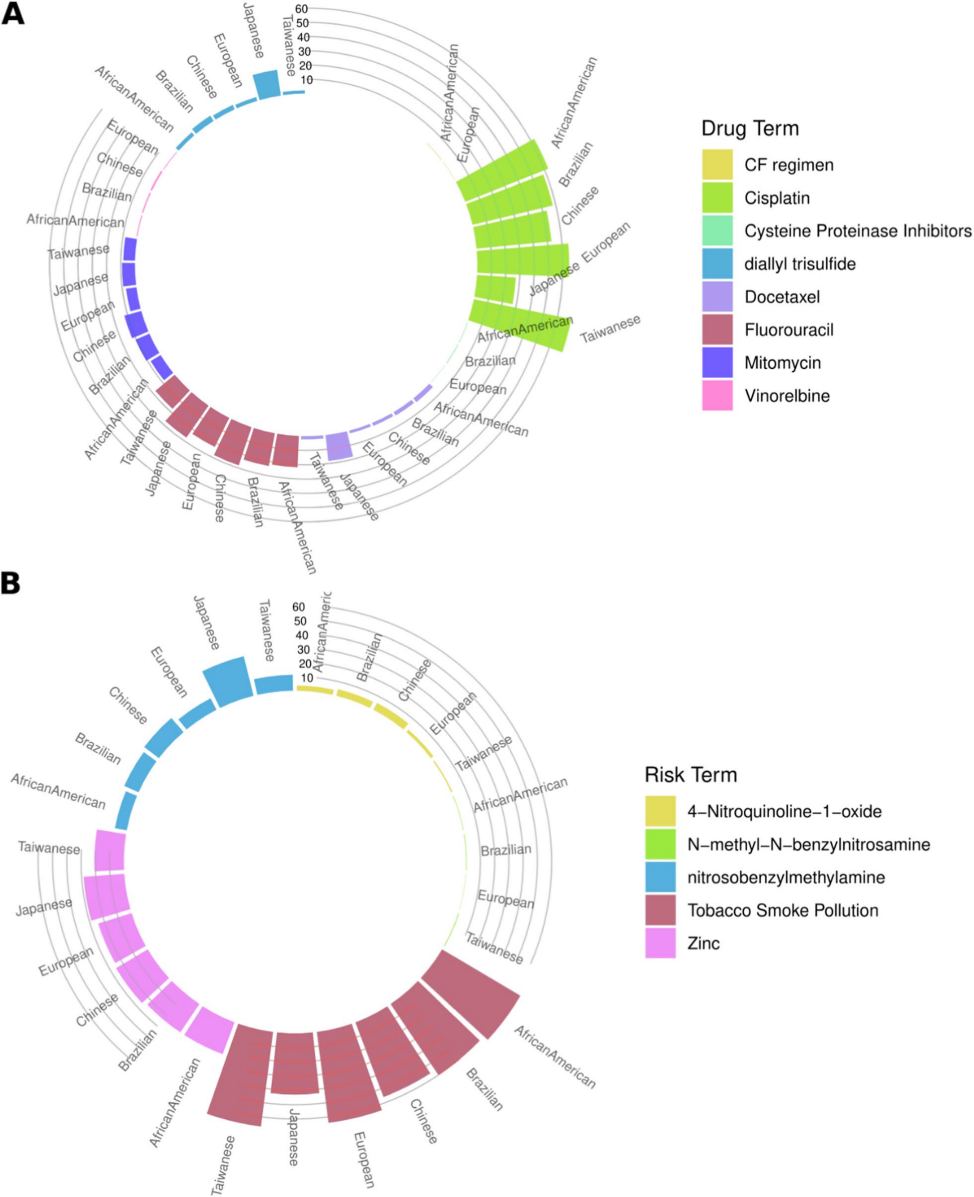
Overrepresented **A** drug chemicals and **B** risk factors interaction among the DEGs inferred from Comparative Toxicogenomic Database

If we considered all the potential associations ignoring evidence for the “direct” associations, we found that ~ 60% of all the DEGs were linked with tobacco smoke across all the populations, however, this was relatively lower in the Japanese population (~ 40%) (Fig. 6A). Similarly, ~ 25% and ~ 10% of DEGs were associated with Zinc and Nitroso Benzylmethylamine across populations, with an exception being Japanese where we found ~ 30% of DEGs were associated with Nitroso Benzylmethylamine. Nitroso Benzylmethylamine is a known carcinogen and mutagen, with the potential to cause cancer and genetic mutations in the cells. The Japanese population showed a higher fraction of DEGs that were associated with Nitroso Benzylmethylamine because of the fact that certain traditional Japanese foods, such as pickled vegetables and fermented fish products, contain high levels of Nitroso Benzylmethylamine [67–69].

We additionally retrieved the gene-drug associations from the CTD. We found the highest fraction of our DEG list was associated with Cisplatin across all populations, followed by Fluorouracil. Both of these chemotherapeutic agents are commonly used for the treatment of ESCC [70, 71]. We once again observed that the DEGs particularly in the Japanese population showed different trends of association with the drugs compared to all other populations (Fig. 6A). We found a relatively low fraction of DEGs associated with Cisplatin, but a higher fraction with Fluorouracil, diallyl trisulfide, and docetaxel. This could be because of the fact that only fluorouracil-based regimens showed higher survival incidences and lower hematologic toxic effects than cisplatin plus fluorouracil-based regimen, in the JCOG9205 trial that was conducted by the Japan Clinical Oncology Group [72]. Docetaxel is another chemotherapy drug that was investigated in a phase II clinical trial, where the tolerability of docetaxel as a single agent in Japanese patients with metastatic esophageal cancer was analyzed [73]. The results showed that the Docetaxel was fairly well tolerated and there were no treatment-related deaths. A higher fraction of DEGs particularly from the Japanese population was also associated with the dietary supplement Diallyl trisulfide (DATS) which is a characteristic flavor component of the essential oil prepared from garlic (*Allium sativum L.*) [74] (Fig. 6A). The antitumor activities of DATs are widely investigated across different types of cancers [75, 76]. The genes inferred for “direct” and “indirect” association with toxicogenomic terms in each population are provided in Additional file 2: Table S9.

## Discussion

We conducted a study where we analyzed microarray datasets of ESCC from six distinct global populations and compared the gene expression patterns. Our analysis revealed that while the majority of the DEGs were consistent across all populations, we identified a small subset of discordant DEGs (19 genes). These genes can provide valuable insights into the genetic and molecular mechanisms underlying the disease, and their discordant expression patterns suggest that population-specific environmental factors, genetic signatures, epigenetic variation and Cis- and Trans-Acting Expression Quantitative Trait Loci [77–79] may contribute to differences in disease progression, drug response, and overall prognosis.

Further characterization of these population-specific gene expression patterns could potentially help identify potential therapeutic targets to certain populations and enable the development of personalized treatment strategies that account for genetic and molecular variations across populations. Additionally, this information could aid in the development of biomarkers to predict drug response or disease prognosis in specific populations, which can ultimately lead to more effective and targeted treatments for patients with ESCC.

We additionally report 166 novel DEGs in ESCC, which is a significant finding. These DEGs have not been previously associated with ESCC and may play a role in the development and progression of the disease. The list of identified genes includes various novel genes as well as some that have previously been implicated in cancer. For example, the upregulation of KLK7, a gene encoding kallikrein-related peptidase 7, has been linked to the promotion of cell proliferation and invasion in several types of cancer [80, 81]. Similarly, the upregulation of DSG1, a gene encoding desmoglein-1, has been implicated in the development of several types of cancer, including head and neck squamous cell carcinoma. Serine aspartate repeat containing protein D (SdrD) of Staphylococcus aureus has been found to directly interact with DSG1 of human squamous cells [82]. It is important to note that DSG2 which is known to upregulate in ESCC has been identified to be a substrate for Helicobacter Pylori HtrA receptor in epithelial cells [83]. Similarly, ENDOU is substrate for Nsp15 of Nido family viruses, which encodes for a protein with endoribonuclease activity that binds to the polyuridine-enriched single-stranded RNA [62]. NSP15 has been reported to be involved in the viral replication process and in the evasion of the host immune system [84]. Although there is limited information on the role of ENDOU in cancer, it has been shown to play a role in other cellular processes such as DNA damage response, stress response, and cell death. ENDOU regulates c-Myc expression by regulating the AICD of B cells [85]**.** The downregulation of ENDOU in ESCC samples plausibly promote tumorigenesis or progression of the disease. However, further studies are needed to elucidate the underlying mechanisms by which ENDOU may contribute to ESCC development and progression. The fact that ENDOU is overexpressed in healthy esophageal mucosa tissues suggests that it may play a role in maintaining the normal function of the esophagus. Understanding the functional role of ENDOU in the esophagus and how it is altered in the context of ESCC could provide valuable insights into the development of novel diagnostic and therapeutic approaches for this disease.

The identification of candidate genes for prognosis in ESCC is an important step towards the development of new biomarkers for early detection and treatment of this cancer. In this study, we identified three novel genes—CHRM3, CREG2, and H2AC6—that showed a significant association with overall survival in ESCC. CHRM3 (cholinergic receptor muscarinic 3) is a novel gene in ESCC, but its expression has been associated with poor prognosis in endometrial carcinoma [86]. Interestingly, CHRM3 has been linked to a well-reported gene KLF4 in ESCC via CHRM3-AS2. A previous report showed that silencing of CHRM3-AS2 expression inhibited cell viability, colony formation, migration, and invasion and promoted apoptosis effects by targeting miR-370-5p/KLF4 in Glioma [87]. This finding suggests a potential role of CHRM3 in tumorigenesis and progression in ESCC. CREG2 (cellular repressor of E1A stimulated genes 2) is highly expressed in malignant gastric cancer tissues and is positively correlated with tumor clinical stage, tumor metastasis, and stages of tumor infiltration. In our study, we found that CREG2 was highly expressed in ESCC, suggesting its potential as a prognostic marker in ESCC. However, GTEx data suggests that CREG2 is not highly expressed in esophageal tissues, indicating that its expression may be tissue-specific [88]. H2AC6 (H2A Clustered Histone 6; alias HIST1H2AC) is moderately expressed in esophageal tissues, but in our study, we found that it was downregulated in ESCC. HIST1H2AC has been shown to be progressively downregulated in HPV-positive neoplastic keratinocytes derived from uterine cervical preneoplastic lesions at different levels of malignancy [89]. This finding suggests that the downregulation of HIST1H2AC may also play a role in the development and progression of ESCC. Overall, these findings provide a foundation for further investigations into the underlying mechanisms of the disease.

Based on our functional enrichment analysis, we identified the Extracellular Matrix related terms were particularly overrepresented in the DEGs across populations. The extracellular matrix is a complex network of proteins and other molecules that provide structural support to cells and play important roles in cell signaling, adhesion, and migration. Alterations in ECM organization and function have been implicated in the development and progression of many types of cancer [90]. Hence, the overrepresentation of ECM-related GO terms and pathway annotations among DEGs in ESCC suggests that alterations in ECM organization may play a significant role in the development and progression of this cancer. ECM changes may contribute to tumor cell invasion and metastasis, as well as alterations in cell signaling pathways that promote tumor growth and survival. Understanding the role of the ECM in cancer development and progression, and targeting ECM-related pathways and molecules may be a promising strategy for developing new treatments that can improve patient outcomes in ESCC.

ESCC is a type of cancer that is commonly associated with chronic inflammation, a characteristic shared by many other types of cancer. As expected, our analysis revealed an overrepresentation of gene ontology terms related to the immune response and signaling pathways among the DEGs in ESCC. Interestingly, we also observed population-specific differences in overrepresented terms. For instance, while the humoral immune response and antimicrobial humoral response were found to be deactivated in Europeans, adaptive immunity was activated and innate immunity was deactivated in Brazilians. The respective human microbiomes may influence these differences in immune response in their geographic regions.

Our analysis also revealed an overrepresentation of pathways related to virus infections among the DEGs, which was not surprising given the well-established links between certain microbial species such as HPV [91], CMV [55, 56] (Table 4), and ESCC. In addition, we also observed an overrepresentation of KEGG pathways related to protozoans such as Amoebiasis and Leishmaniasis in our analysis, which is an interesting finding because both of these infections are prevalent disease throughout tropical and subtropical regions of the world. In this line we also identified genes associated to CMV and Leishmaniasis were enriched in Brazilian samples, highlighting potential population and environment-specific differences in the etiology of ESCC.

Our investigation revealed a consistent link between tobacco smoking and ESCC across various populations. The smoke from tobacco contains harmful chemicals such as nitrosamines and polycyclic aromatic hydrocarbons, which can cause DNA damage and increase cancer risk. ESCC patients who smoke also tend to have more advanced disease stages and a higher likelihood of recurrence and mortality than non-smokers. Moreover, smoking may interfere with the effectiveness of various ESCC treatments such as chemotherapy, radiation therapy, and surgery. Our study also identified Cisplatin and Fluorouracil as commonly used drugs for treating ESCC across different populations. We further examined genes associated with the response to these drugs. Interestingly, we observed a higher fraction of DEGs linked to the dietary supplement Diallyl trisulfide, a component of garlic oil, in the Japanese population. This compound has been extensively studied in Japan for its anti-tumor properties.

## Conclusion

In conclusion, this study provides an analysis of DEGs across 12 datasets of ESCC in different populations. By filtering against ESCC ATLAS, we identified 1442 DEGs, including 1423 concordant DEGs and 19 discordant DEGs. Among the 1423 concordant DEGs, we found 1257 mapped to ESCC ATLAS, and some interesting genes identified included SASH1, which was downregulated in multiple populations, and BLNK, which was downregulated in all populations except the Japanese. We also identified 166 novel DEGs, of which 19 showed the exact opposite expression trend in healthy esophageal-mucosa, indicating their importance in ESCC. This study highlights the differences in DEG expression across different populations and genomic landscaping of microbial connections including Nido family viruses, HPV, Entamoeba histolytica, Lieshmainia, and staphylococcus aureus provides novel insights into the coinfection in etiology of ESCC. Further research could investigate the functional roles of these DEGs in the pathogenesis of ESCC, potentially leading to the development of precise targeted therapies for this disease.

### Supplementary Information

**Additional file 1 Fig. 1** Workflow followed in the data analysis. **Fig. 2** QQ-plot showing observed versus theoretical quantiles of expression in all the 12 ESCC ‘normal vs tumor’ data-sets

**Additional file 2 Table 1** List of identified DEGs (1432) in ESCC. **Table 2** List of Novel DEGs (166) identified in ESCC. **Table 3** List of Discordant DEGs (19) identified in ESCC. **Table 4** List of Novel discordant DEGs (6) found in our ESCC analysis. **Table 5** List of overrepresented Gene Ontology (GO) terms in ESCC. **Table 6** List of overrepresented KEGG pathways in ESCC. **Table 7** List of overrepresented REACTOME pathways in ESCC. **Table 8** List of overrepresented Wiki pathways in ESCC. **Table 9** List of genes found connected to Toxicogenomics terms in ESCC

## Data Availability

The data generated in this study are available within the article and its supplementary data files.
