# Supplementary material for: Global comparative transcriptomes uncover novel and population-specific gene expression in esophageal squamous cell carcinoma
Source: Infect Agent Cancer. 2023 Aug 28;18:47. doi: 10.1186/s13027-023-00525-8 (PMC10463703; doi:10.1186/s13027-023-00525-8)

Supplementary Figure 1: Workflow followed in the data analysis
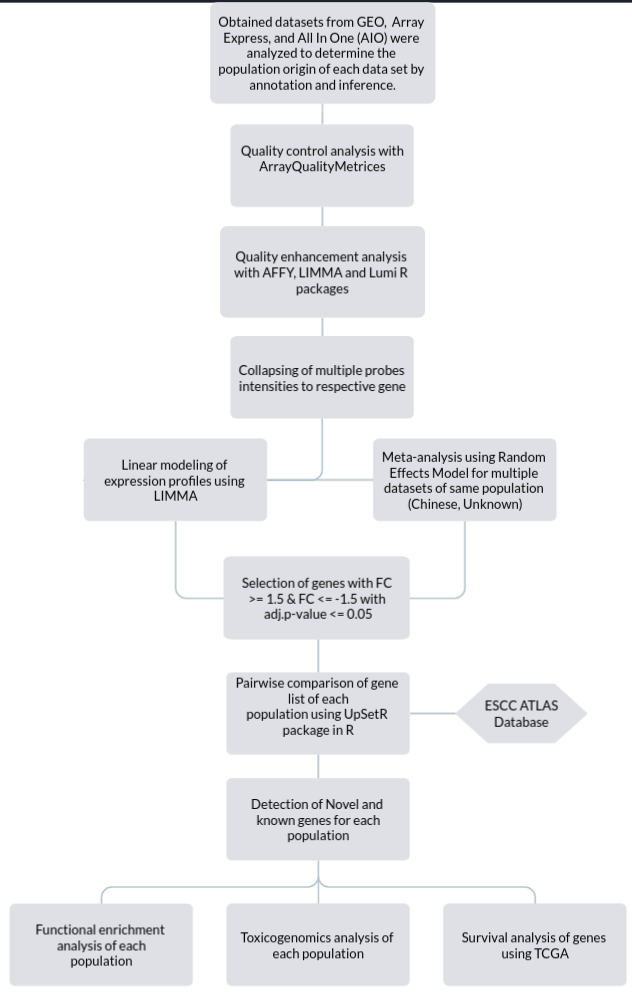


Supplementary Figure 2: QQ-plot showing observed versus theoretical quantiles of expression in all the 12 ESCC ‘normal vs tumor’ data-sets
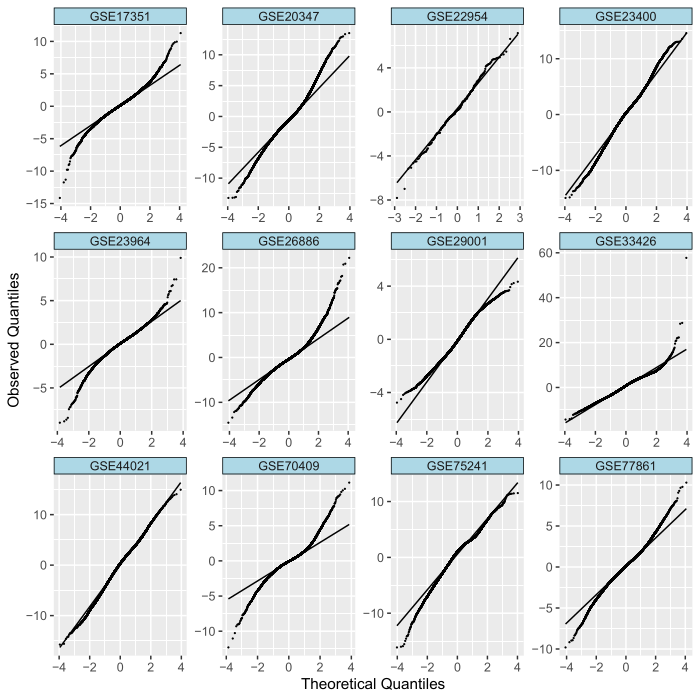

Supplement: Supplementary file 1 — Additional file 1 Fig. 1 Workflow followed in the data analysis. Fig. 2 QQ-plot showing observed versus theoretical quantiles of expression in all the 12 ESCC ‘normal vs tumor’ data-sets [file 13027_2023_525_MOESM1_ESM.docx]
